# Designing Online and Mobile Diabetes Education for Fathers of Children With Type 1 Diabetes: Mixed Methods Study

**DOI:** 10.2196/13724

**Published:** 2019-08-06

**Authors:** Anastasia Albanese-O'Neill, Desmond A Schatz, Nicole Thomas, Jay M Bernhardt, Christa L Cook, Michael J Haller, Angelina V Bernier, Janet H Silverstein, Sarah C Westen, Jennifer H Elder

**Affiliations:** 1 College of Medicine University of Florida Gainesville, FL United States; 2 Moody College of Communications University of Texas at Austin Austin, TX United States; 3 College of Nursing University of Central Florida Orlando, FL United States; 4 Department of Clinical and Health Psychology University of Florida Gainesville, FL United States; 5 College of Nursing University of Florida Gainesville, FL United States

**Keywords:** type 1 diabetes, mobile health, fathers, stakeholder participation

## Abstract

**Background:**

Fathers make unique and central contributions to the health of their children. However, research in type 1 diabetes (T1D) education largely ignores the needs of fathers, including during the development of online and mobile educational materials.

**Objective:**

The purpose of this study was to solicit and incorporate input from fathers of children with T1D into the design, content, and infrastructure of a suite of online diabetes self-management education and support (DSMES) resources.

**Methods:**

The study took part in three phases: (1) exploratory research, (2) website and subdomain development, and (3) evaluation. Fathers of children with T1D (n=30) completed surveys and semistructured qualitative interviews. Thematic content analysis was used to identify fathers’ content and design preferences. An online DSMES website (T1DToolkit.org) and a separate mobile subdomain targeting fathers (Mobile Diabetes Advice for Dads, or mDAD) were developed. A prototype of the site for fathers was evaluated by 33 additional father participants. End user feedback was elicited via survey.

**Results:**

Participants in the exploratory phase were enthusiastic about the online diabetes resources. Preferences included high-quality design, availability via mobile phone and tablet, brief text content supplemented with multimedia and interactive features, reminders via text or email, endorsement by medical professionals, and links to scientific evidence. The mDAD subdomain received high usability and acceptability ratings, with 100% of participants very likely or likely to use the site again.

**Conclusions:**

The development of eHealth educational platforms for fathers of children with T1D remains an unmet need in optimizing diabetes management. This study incorporated fathers’ feedback into the development of a suite of online diabetes education resources. The findings will serve as the basis for future research to assess the clinical efficacy of the website, its subdomain targeting fathers, and additional subdomains targeting unique populations.

## Introduction

Type 1 diabetes (T1D) is one of the most commonly diagnosed chronic childhood conditions. Despite significant advancements in glucose monitoring and insulin delivery technology over the past two decades, only a small percentage of children with T1D in the United States meet glycemic targets set forth in clinical guidelines [1-2]. In 2016, the American Academy of Pediatrics published its second clinical report on fathers, emphasizing the unique and central contributions they make to the health of their children and highlighting the cultural and structural barriers fathers encounter as caregivers [3]. Fathers’ engagement in their children’s T1D management has been linked to improved glycemic outcomes for the child with T1D and improved quality of life for all family members [4]. However, fathers of children with T1D are less likely than mothers to attend clinic visits with their children. As such, fathers are often absent when diabetes education is provided and likewise often miss opportunities to participate in research [5-6]. Few studies have targeted fathers’ diabetes-specific educational needs, and there remains no tailored mobile or Web-based diabetes education available to fathers [7].

The available data reveal that fathers’ knowledge trails that of mothers, fathers report insufficient knowledge to avoid common errors, and fathers’ retention of diabetes knowledge diminishes over time [8-9]. Mothers and fathers alike have reported never mastering diabetes management skills and express a gap between desired knowledge and actual competencies [10]. While health care providers often focus on the mechanics of disease management, in particular glucose control, families indicate a need for assistance in responding to diabetes life challenges, adaptation to the diagnosis, and linkage to social support [11]. Fathers of children with T1D have reported diverse learning needs, with fathers of teenagers indicating more substantial knowledge requirements and skills training to guide their involvement in their children’s care [7,11-14]. However, qualitative studies suggest that fathers can feel lost or left out during clinical appointments because they lack the disease-specific knowledge, vocabulary, or conceptual framework to confidently participate in the conversation with the care provider [15].

Paradoxically, as children become teens and fathers’ diabetes-specific educational needs increase, paternal attendance at clinical visits decreases, falling to 14% of visits for fathers of children ages 13 to 17 years [5]. This is due in part to cultural and health care system barriers such as lack of time off and inconvenient clinic hours, but research also suggests that the diabetes care team may inadvertently discount paternal attendance at clinic visits [3,16]. Fathers themselves may not perceive clinic visits as a positive learning environment or opportunity to improve adaptation to and engagement with their children’s diabetes management [17]. Regardless of the cause, the decline in paternal attendance during adolescence has been correlated with a longitudinal decline in regimen adherence and glycemic control, particularly if the child has low perceived diabetes-specific self-efficacy [18-19]. Collectively, these studies suggest that clinics might not be the best place to provide Diabetes Self-Management Education and Support (DSMES) for fathers. It remains imperative, however, to enhance fathers’ knowledge and involvement in their children’s care to mitigate the decline in diabetes self-care behaviors and glycemic control that typifies adolescence with T1D [1,20].

Parents of children with T1D search for information online [21]. As of October 2018, a search for “diabetes education” in the Google search engine identified over 239 million results and entering “diabetes” into the iTunes App Store search field yielded over 1000 unique results [22]. Despite these voluminous options, there is only a small amount of scholarship in the scientific literature to describe how these diabetes education websites or apps were developed or if their effect on clinical and psychosocial outcomes has been validated [23]. Recent studies have solicited end user feedback to inform other facets of diabetes care, including the development of Web-based decision aids, coping skills training, eHealth behavioral programs, and the use of simulation in diabetes education [24-28]. A particularly promising study evaluating an online coping skills training program has shown improved clinical outcomes [29]. However, many pilot studies using mobile phone and text messaging to assist with diabetes management, track blood glucose, and provide motivation that have shown preliminary feasibility and acceptability have lacked sufficient sample sizes, have had brief intervention duration, or have had other flaws, limiting their generalizability [23,30].

To summarize, fathers of children with T1D have unmet educational needs, and traditional, clinic-based diabetes education delivery may be unsuccessful in meeting these needs due to low clinic attendance [5,7]. Leveraging mobile technology to deliver DSMES to fathers outside of the clinical setting is an important step toward improving their self-efficacy with regard to their children’s care. Currently available diabetes-related mobile apps and websites have not incorporated fathers’ feedback. The purpose of this study was to solicit end user input from fathers of youth with T1D, incorporate their preferences into the design and content of a DSMES website and prototype subdomain targeted to fathers, and beta-test the subdomain with fathers of youth with T1D to ascertain preliminary usability and acceptability.

## Methods

### Design and Methods by Phase

This multiyear, mixed-methods study was conducted in three phases by an interdisciplinary team including certified diabetes educators (CDEs), pediatric endocrinologists, nurse practitioners, dietitians, psychologists, Web designers, graphic designers, videographers, artists, parents of children with T1D, and people living with T1D. The study design was informed by the small but growing field of literature on best practices in eHealth tool development [23-29, 31-34]. In phase 1, data were collected via one-on-one semistructured interviews and an online survey from fathers of children with T1D. In phase 2, the DSMES website (www.T1DToolkit.org) and subdomain targeting fathers (mDAD: Mobile Diabetes Advice for Dads) were developed. In phase 3, participants evaluated the mDAD prototype and provided feedback via survey. All phases of the study were approved by the University of Florida institutional review board, and informed consent was obtained from all participants prior to participation. The study team conducted recruitment, obtained informed consent, and performed data collection and related analyses.

### Phase 1: Exploratory Research

Fathers of children aged 6 to 17 years with T1D were recruited from the University of Florida pediatric diabetes clinic, at JDRF (formerly the Juvenile Diabetes Research Foundation) events, and via flyer. Participants completed surveys that included closed and open-ended questions and took part in a semistructured, one-on-one interview. All surveys and interviews were conducted in English. The quantitative survey results, published elsewhere [7], revealed that fathers of children with T1D had unmet diabetes education needs, including on topics in basic management such as managing hyperglycemia and hypoglycemia and calculating insulin doses, and more nuanced knowledge requirements, including managing diabetes at school, information on emerging diabetes technologies, and finding help for diabetes challenges. There was a high level of interest in online and mobile access to diabetes education resources [7]. The semistructured interview guide (Multimedia Appendix 1) was designed to elicit detailed information on the educational needs and design preferences of participants. Each participant was offered a $40 gift card at the completion of the survey and interview.

Interviews were digitally recorded and immediately transcribed by a professional transcription service. Transcripts were reviewed for accuracy, and names and other identifiers were removed. The content analysis of the responses to open-ended survey questions and interview transcripts began immediately following the first interview and proceeded until saturation was reached. Thematic content analysis was used to characterize participant knowledge requirements and identify benchmark product preferences for the mobile diabetes education website and tailored subdomain. Transcripts were initially coded line by line by two study team members, and these codes were consolidated into themes [35,36]. As themes emerged, the data were reviewed in an iterative process to reach consensus by study team members and the university’s Qualitative Data Analysis Group, a group of faculty and graduate students with expertise in qualitative research methods in health science research who provide consultation and review on qualitative projects.

### Phase 2: Website and Mobile App Development

**Figure 1:**
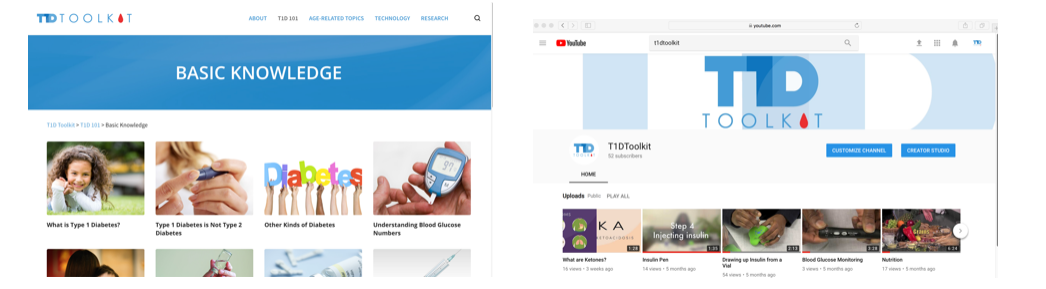
T1D Toolkit website and YouTube channel.

The curriculum from a recognized diabetes education program affiliated with a university-based pediatric diabetes clinic served as the baseline website content. The curriculum covers basic management topics, including pathophysiology; insulin therapy (injection and pump); glucose monitoring, glucose targets, and pattern management; management of hyper- and hypoglycemia; emergency Glucagon administration; sick days and ketone monitoring and treatment; exercise and activity; nutrition and meal planning; social support and psychosocial aspects of care; safety; insurance coverage and financial support; managing diabetes at school; diabetes technology basics; and screening and prevention of chronic diabetes complications. In addition to the study team, the website design team comprised 5 professionals who worked on a part-time/contractual basis and included a Web designer, graphic designer, artist, and videographer to assist with design, animations, and video production. The overall site design was informed by user-centered and sociotechnical design principles [37]. This approach weighs the characteristics and preferences of the product’s target population and integrates user perspectives into product design to increase usability and acceptability. Exploratory findings were used to develop and refine the DSMES website, www.T1DToolkit.org (Figure 1).

Based on guidance from the American Medical Association and National Institutes of Health, content on the website and subdomain was written at the 6th grade level; readability was confirmed by separate analysis [38]. All content was presented in English. A video or narrated animation was developed for each major educational topic or skill to ensure accessibility for users of all literacy levels. This necessitated the creation of a YouTube channel to house the video content, which could then be easily embedded and cross-purposed within various pages/posts on multiple sites/subdomains as desired. The YouTube channel is branded with the T1D Toolkit logo. Its content can only be accessed via password and the channel is routinely monitored by the study principal investigator. Push technology capability (the ability to push links to educational material to the end user via text or email) was facilitated via inclusion of a plug-in to automate this process. Content was informed by the phase 1 qualitative analysis and quantitative data on fathers’ educational needs and technological preferences reported elsewhere [7].

**Figure 2:**
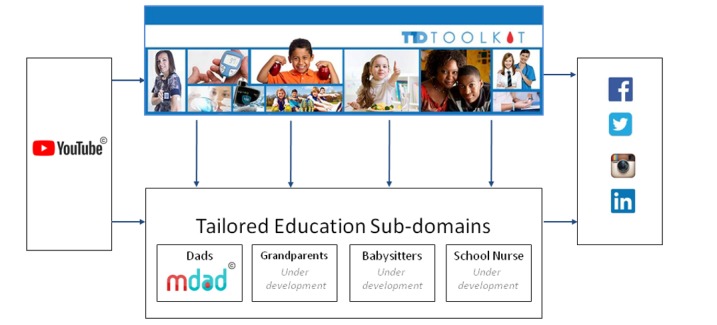
T1D Toolkit online architecture.

The DSMES site content was organized into the following categories: (1) T1D 101 (topically sorted into basic skills, basic knowledge, support and connections, newly diagnosed, diabetes definitions); (2) Age-Related Topics (topically sorted into developmentally specific information for children with T1D during preschool, school age, teens, and young adult years); (3) Technology (topically sorted into glucose monitoring, insulin delivery, emerging technology, living with technology); and (4) Research. This grouping of topics reflects exploratory findings as well as iterative feedback obtained during website development. The site was designed to serve as a library for the subdomain targeted to fathers, with the capacity to support other tailored subdomains for babysitters, grandparents, siblings, school personnel, etc. T1D Toolkit–branded social media platforms were established on Facebook, Instagram, Twitter, and LinkedIn to facilitate dissemination of information and increase reach. The T1D Toolkit online infrastructure with current and potential subdomains can be seen in Figure 2.

Video scripts were written by a clinical website development team including nurses, registered dietitians, CDEs, nurse practitioners, and physicians, reviewed by medical personnel, and then recorded, postproduced, and rendered. When possible, members of the diabetes care team, children/adults with diabetes, or parent caregivers were featured in the videos to improve relevance and create an opportunity for peer learning. It was agreed that the site would remain free from commercial influence to enhance trustworthiness. A “Not what you were looking for?” link was added to each page that could be completed by visitors who did not find the information they sought.

**Figure 3:**
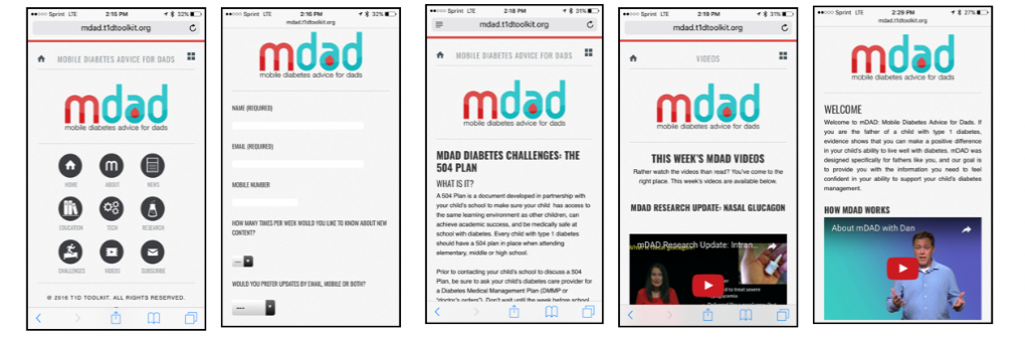
Screenshots of subdomain mDAD: Mobile Diabetes Advice for Dads.

The mDAD subdomain’s content is drawn from the DSMES website but can be rebranded specifically for the target audience. The architecture of this subdomain reflected the priorities identified by father participants in the exploratory phase, with an emphasis on delivering information about diabetes technology and T1D research findings. Screenshots of the mDAD subdomain optimized for mobile devices can be seen in Figure 3. Separate logos were developed for the mDAD site and YouTube channel. The mDAD subdomain is a mobile website optimized for handheld devices such as mobile phones and tablets; it is not an app. Unlike apps, a mobile website can be kept up to date by the developer and does not rely on the end user to download updates. Moreover, mobile websites do not use data storage space on the end user’s mobile device and do not collect identifying information from the end user. The mDAD subdomain has its own mDAD-branded social media platform.

### Phase 3: Evaluation of the Mobile Diabetes Advice for Dads Subdomain’s Usability and Acceptability

Thirty-three fathers of children with T1D who did not participate in the exploratory phase were recruited during their attendance at the Children with Diabetes Friends for Life Conference in Orlando, FL, to evaluate the mDAD subdomain (mDAD.T1DToolkit.org). Participation was voluntary, and no compensation was provided. Following consent, participants were advised to explore the mDAD site on a mobile device (tablet or mobile phone) and then complete an anonymous survey that included 18 closed-ended and 2 open-ended questions about the site’s usability, design quality, and content. Responses to closed-ended questions were measured using a 5-point Likert-like scale, with a total possible 90 points; higher scores indicated higher levels of usability (ability to access the site, review content, and navigate the site), and acceptability (quality of content and design). Descriptive statistics were generated using SPSS Statistics (IBM Corp) software.

## Results

### Phase 1: Exploratory Research

Thirty fathers of children aged 6 to 17 years with T1D participated in phase 1 of the study (Table 1). The mean age of fathers was 47.7 (SD 8.5) years, mean child age was 12.5 (SD 3.4) years, and mean diabetes duration was 6.5 (SD 4.1) years. Analysis of the qualitative interview transcripts revealed three themes that would guide the development of the online diabetes education resources.

**Table 1.** Demographics of study participants (N=30).

Characteristics		n (%)
**Race/ethnicity**		
	Black/African American	2 (7)
	Hispanic/Latino	2 (7)
	White	25 (84)
	Asian	0 (0)
	Other	1 (3)
**Highest education obtained**		
	Some high school	0 (0)
	High school graduate/General Education Development	4 (13)
	Some college but no degree	4 (13)
	Associate’s degree	1 (3)
	Bachelor’s degree	10 (30)
	Master’s degree	6 (20)
	Professional degree or doctorate	6 (20)
**Household income**		
	Less than $29,999	1 (3)
	$30,000 to $49,999	2 (7)
	$50,000 to $74,999	4 (13)
	$75,000 to $99,999	3 (10)
	$100,000 to $149,999	6 (27)
	Over $150,000	12 (40)
**Employment status**		
	Employed, full-time	27 (90)
	Employed, part-time	2 (7)
	Retired	1 (3)

#### Theme 1: Perceived Value of Online Diabetes Education Resources

Overall, participants expressed a high level of acceptability for receiving diabetes education online, particularly via mobile phone or another mobile device. Of particular interest were the ability to review information at their own pace, convenience of not having to be in a fixed location, and freedom from judgment conferred by a private learning environment. A minority of participants noted they preferred in-person/clinic or group-based diabetes education. While all participants said they made the effort to attend clinic visits with their children, many were unable to do so due to scheduling conflicts. A positive relationship with their diabetes care team was nearly universal; however, some participants indicated that routine appointments were not a venue where problems were solved or new information was obtained, and others noted they sometimes felt uncomfortable asking questions during clinical visits and/or worried they were being judged for their lack of knowledge. For fathers who did not attend visits, some viewed the child’s mother as becoming a gatekeeper for diabetes knowledge and disease management. One father who was able to attend only some of the visits put it this way, “I hate it when she goes to the doctor and I don’t because I don’t like feeling like I didn’t hear the whole story.” Fathers of female adolescents noted that both they and their daughters felt uncomfortable talking about puberty-related topics that invariably came up at visits (menstruation, birth control, physical development, etc), perhaps contributing to a decline in paternal attendance. A summary of the benefits of online diabetes education enunciated by father participants, along with illustrative quotes, can be found in Textbox 1.

**Fathers’ perceived value of online diabetes education resources.**

On my time:

- “Online is, obviously, easier. Online, where you can jump in on your schedule, and jump out on your schedule is better for me.”

Lower perceived value in clinic visits:

- “The doctor visits aren’t solution based. They’re just visits to see that you’re managing it.”
- “I know—I know for a fact, after talking with a lot of other parents, that they are reluctant to ask that question, for fear of sounding or looking dumb, so they let it go.”

Concerns about judgment:

- “I don’t know as much about it as I should know about it. I already feel crappy about that. I didn’t want to go and interface in a set of circumstances where I’m going to feel like somebody’s being judgmental about what I don’t know.”

Learning independently:

- “I’m more focused on learning on my own.”
- “I’ve been through too many groups, and they get so easily distracted that it becomes a lot of—a lot of crybabies and whining. I don’t like groups.”
- “For me, group settings just drain the energy out of me.”

#### Theme 2: Website and Subdomain Content Priorities

Although the average diabetes duration of the participants’ children was 6.5 (SD 5.1) years, all participants noted a need to review basic diabetes knowledge and skills, particularly those that they did not use routinely. They advised that online information should be organized by age and tailored to the child’s developmentally specific diabetes needs (eg, toddler versus teenager versus young adult). They sought guidance during developmental transitions often complicated by diabetes, including starting school, middle school, puberty, driving, leaving home, and going to college. They asked for expert advice on current and emerging diabetes technologies; not only what was available but how to use it effectively (eg, information about brands and models of currently available continuous glucose monitors and guidance on how to access and interpret continuous glucose trend data). All father participants in the study desired information on current research that was presented in a way they could understand, and many looked for information about risks for chronic diabetes complications, had questions around health insurance, and worried about diabetes discrimination. Participants indicated they wanted to choose topics specific to their individual interests. As one participant explained, “I don’t think just getting random bits of information that may or may not be tailored to somebody’s specific, individual needs is particularly helpful.” A summary of the desired content priorities identified by father participants, along with illustrative quotes, can be found in Textbox 2.

**Website and subdomain content priorities.**

Basic management:

- “It would be nice to have answers that were easily accessible to the kinds of questions that I have, like, [chuckles] what is a ketone? Why should I care? What the hell should I do about it?”
- “It’s more just needing just basics...”

Organized by age:

- “Break it into the different stages, your child, teen, adult.”
- “Different age groups, what’s good for toddlers? What’s good for growing young boys and girls to teenagers?”

Guidance on transitions:

- “These transitions. Middle school, high school, driving.”
- “Puberty, what’s puberty going do to him?”
- “It’d be nice to have—to be able to ask other families how they’ve dealt with the diabetes once their kid goes to college.”
- “I have to let go. She’s going to a college 100-plus miles from home.”

Technology:

- “I would want to know more about the technology, number one...”
- “If I had something that...—was a gold standard that I could turn to and get the state of the art on continuous glucose monitor or the best meter or the best pump or what to try, what to stay away from.”

Research:

- “I’d like to know more about the research and what’s going on and long-term to find a cure, and what the different types of approaches that they’re looking at.”

Other:

- “Long-term health and insurance coverage.”
- “Is there any discrimination against diabetics? Are people going to treat my daughter differently because she’s diabetic?”

#### Theme 3: Design Recommendations

Participants clearly enunciated their design preferences, prioritizing high quality design and multimedia features including a combination of videos, images, animations, illustrations, interactive features, links to other resources/evidence, infographics, and downloadable reference sheets (PDF). Many participants acknowledged that brief videos would be optimal to convey information, particularly for fathers with lower educational attainment. Video content was also valued regarding skills training, especially for invasive or emergency skills. As one father noted, “I don’t think that without visualization you can really apprehend what it is that you’ve got to do.” Participants repeatedly stated that the information provided should be brief and concise, contain links to more in depth information and evidence, should be curated by the diabetes care team, and should be pushed to them in a structured way via email, text, or social media. There was an underlying concern that the information should be trustworthy, up to date, free of commercial influence, and reflect evidence rather than opinion. One father commented, “Once I learned facts versus seeing only horror stories online, I steered away from looking online at stuff.” Most fathers said that they would be more likely to trust online information if it were endorsed by medical experts, had a direct connection to clinical practice, or was recommended by the child’s pediatric endocrinologist or CDE. A summary of the design priorities identified by father participants, along with illustrative quotes, can be found in Textbox 3.

**Design recommendations.**

Videos and animations:

- “For skills like administering glucagon, I don’t think that without visualization you can really apprehend what it is that you’ve got to do.”
- “Being in education though, especially for people who are visual and who are not able to read and write, [video] is so important. Because we assume that people can read and write, which they can’t.”
- “I'm a visual learner. Obviously, I think YouTube is an awesome medium for learning.”

Concise:

- “Things that are targeted. Bullet points, as opposed to long articles.”
- “I’m better in smaller doses because I don’t have a lot of time.”

Push technology:

- “If it’s new information or updated information—I would want a reminder or a notice.”
- “I would like the idea of pushing it to us rather than going and looking for it because that’s sometimes complicated.”

Up to date:

- “Something you could make sure you’re on top of everything and you’re up to the latest on all the diabetes news that’s coming down the line.”
- “Of course, as you know with diabetes, the information and the—it’s changing that [snaps fingers] quick.”

Mobile:

- “I like it on my phone, because I pretty much have that with me everywhere. I look at it more if it’s on my phone. If it’s on the computer, it’s not as convenient.”

Credible, endorsed, attractive:

- “I think an app’s a great idea. Everybody wants everything at their fingertips. I think the important thing is how it’s laid out, how did they get the information, and can the dad trust it?”
- “Then, they’ve also got to be right—because there’s so much misinformation or partial information. You want to know the answer to your question, and you want it to be actionable, where the response is accurate.”
- “That the information’s accurate, that the information is true.”
- “If you’ve got a poorly designed webpage covered with advertisements, I’m not likely to trust it.”

### Phase 3: Evaluation of the Mobile Diabetes Advice for Dads Subdomain’s Usability and Acceptability

Thirty-three fathers of children with T1D who did not participate in phase 1 of the study were enrolled in phase 3 to evaluate the mDAD subdomain. The phase 3 feedback survey had a potential range of 18 to 90 points. Phase 3 participants reported high levels of usability and acceptability, with an average score of 85.6 points (range 78 to 90 points). All participants reported they would be very likely or likely to visit the site in the future; 97% (32/33) rated the overall quality of the site as excellent or very good; 91% (30/33) reported they strongly agreed or agreed that the information on the site was useful to them; 97% (32/33) strongly agreed or agreed the site was well organized; 100% (33/33) reported the overall layout and design of the site was attractive; 85% (28/33) reported they were always able to view the videos; and 91% (30/33) reported they never or rarely got lost when looking for information. The results of the product evaluation can be found in Multimedia Appendix 2.

## Discussion

### Principal Findings

Few eHealth apps or websites solicit and incorporate end user perspectives into design and content decisions, and fewer still are validated by a rigorous design and development process. This study represented a first step in incorporating end user feedback into the design and content of a suite of online diabetes education resources for children with diabetes and their parent caregivers. Exploratory phase feedback was used to develop the online diabetes education resources www.T1DToolkit.org and mDAD.T1DToolkit.org. In the evaluation phase, participants reported high levels of acceptability for mobile delivery of diabetes education and skills training. This study is particularly novel because it demonstrated it was possible to successfully recruit father participants, thereby facilitating meaningful input from a population that is typically left out of pediatric research. Of note, recruitment largely took place outside of the clinical setting where the accompanying parent is often the mother, suggesting that paternal recruitment may be more successful when conducted at diabetes conferences, via flyers, and through diabetes volunteer organizations.

As noted above, mothers have typically been the parent most directly involved in their children’s care after a child’s diagnosis with T1D. Likewise, mothers have most often participated in diabetes education research, and maternal feedback has been used to inform the diabetes education process and related resources over time. However, fathers’ engagement in their children’s T1D management has been correlated with improved adherence, better psychosocial adjustment, and improved overall family health, and fathers are becoming more involved in their children’s care [3,18-19,39-42]. As such, fathers should be welcomed as integral diabetes caregivers as recommended by the American Academy of Pediatrics [3]. Creating a paradigm of care to help fathers avoid cultural or systemic barriers (eg, inconvenient office hours, lack of direct access to diabetes education, mother as gatekeeper) to participation in their children’s care should be a priority for pediatric diabetes practices. Solutions to this problem include online education and skills training, flexible office hours, and the use of telemedicine to augment traditional clinic visits. Fathers who cannot attend clinic visits should have direct access to diabetes education, which necessitates the development and availability of validated online resources. Diabetes education that is available online or via mobile technology improves paternal access to diabetes knowledge and skills training and mitigates the gatekeeper role of mothers. This study contributes to our understanding of how to develop and sustain validated online and mobile diabetes education resources available outside of the clinic setting.

Although there is a low level of trust in the information found online, parents of youth with T1D report frequent use of the internet and online diabetes forums to acquire knowledge [20]. Our study confirms earlier findings that parental trust in online information about diabetes is enhanced by a commercial-free environment, links to evidence, and endorsement by an accredited diabetes clinical team [43]. Not only did our participants enthusiastically support the delivery of diabetes education via mobile and push technology, they cited unanticipated benefits, including the opportunity to acquire knowledge in a safe, private environment without fear or embarrassment. This concept of empowerment derived from the use of eHealth tools has been recommended as an important consideration in the development of T1D apps in systematic reviews [32].

Participants in our study also identified shifting priorities associated with their children’s evolving developmental imperatives, underscoring the complexity of parenting a young child with T1D through adolescence into adulthood and the need for guidance on how to cope with the day-to-day challenges presented by T1D in real-life situations [11]. Our participants repeatedly expressed the need for anticipatory guidance specifically related to their children’s developmental stage and life transitions, including attending school for the first time, going through puberty, surviving middle school, going to college, and moving away from home. The importance of social support and the opportunity for peer learning should be strongly considered when educational interventions, both online and in person, are developed.

Diabetes education is poorly reimbursed and typically only provided comprehensively at diagnosis. In addition, families may not attend clinic-based education-focused visits because they are not a covered service and are costly to the family, require additional time off from work or school, or require the family to travel a long distance at a personal cost. The ubiquity of mobile phone technology makes the possibility of online diabetes education a reality, but only if it is presented in an accessible way and with reliable scientific evidence to support specific recommendations.

### Limitations

This study had several limitations. All fathers and stepfathers in the exploratory phase lived with their children in two-parent homes, and the majority had obtained a college degree. In addition, demographic data were not collected during the beta test. Additional research is required to ascertain the learning needs of minority fathers, single fathers, same-sex male couples, and fathers with lower incomes and less formal education. The usability and acceptability measures used in the study were not standard tools for assessing these constructs; future studies should use a standard usability tool in addition to study-specific measures. Observational data regarding how participants navigated and viewed the site were not collected. Finally, despite the use of rigorous processes to limit bias during qualitative data analysis, there still exists the inherent risk that study investigators may not have effectively bracketed their preexisting presuppositions.

### Conclusions

The publicly available website, www.T1DTookit.org along with its subdomain targeted to fathers and future subdomains targeted to additional audiences have the potential to complement and supplement diabetes education provided in the clinical setting, with the ultimate goal of contributing to improved diabetes outcomes. The parent website has been integrated into the clinic’s diabetes education paradigm, and the subdomain targeting fathers is being modified based on feedback obtained in this study. Future studies will solicit input from a diverse sample of end users to refine the suite of eHealth online resources. This will be followed by a clinical trial to determine the potential of these resources to contribute to improvements in the glycemic control of children with T1D and improvements in diabetes knowledge and psychosocial outcomes for all family members, including those who cannot routinely attend clinic visits. The promise of technology to reduce cost and improve diabetes outcomes will only be realized by placing evidence behind mobile diabetes websites and apps that can then be easily and cost-effectively incorporated into clinical practice.

## Appendix

Multimedia Appendix 1 Qualitative interview guide.

Multimedia Appendix 2 Survey responses for usability and acceptability of the Mobile Diabetes Advice for Dads subdomain.

## Abbreviations

CDE: certified diabetes educator
DSMES: Diabetes Self-Management Education and Support
mDAD: Mobile Diabetes Advice for Dads
T1D: type 1 diabetes
