# Supplementary material for: Designing Online and Mobile Diabetes Education for Fathers of Children With Type 1 Diabetes: Mixed Methods Study
Source: JMIR Diabetes. 2019 Aug 6;4(3):e13724. doi: 10.2196/13724 (PMC6701161; doi:10.2196/13724)
Supplement: Multimedia Appendix 1 [file diabetes_v4i3e13724_app1.pdf]

Phase I Interview Guide

1. Tell me about your family.

    a. Tell me about your child with diabetes.

2. How has life changed since your child was diagnosed with diabetes?

3. How are you dealing with diabetes within your family? Please describe a good day with diabetes? What about a bad day?

4. What parts of diabetes do you feel most comfortable with? Least comfortable?

    a. How do you help your child with diabetes?
    b. How does your family manage diabetes?
    c. Who goes with your child to diabetes appointments?

5. Are there parts of diabetes management that you would like to learn more about? If so, what are they?

6. Describe the ideal way for you to get answers to questions you have about diabetes.

    a. What do you think about videos or games to help you learn more about diabetes?
    b. Would you prefer to learn in a group setting? Why or why not?
    c. Would you like to learn a lot all at once or a little each day? Why?

7. Is there anything you think I've missed that is important to know about what it's like being a dad who cares for a child with diabetes?
f
    a. Is there anything else we haven't talked about today that I should know about the best way to provide diabetes education for dads/stepdads?

---

9. Does anyone else in the family have diabetes?

10. What kind of insulin does your child currently take?

Insulin name(s):

How does your child take insulin?

Vials and syringe Pens Pump Combination of methods

What are your child's insulin doses?

11. Does your child count carbohydrates?
