# Supplementary material for: Designing Online and Mobile Diabetes Education for Fathers of Children With Type 1 Diabetes: Mixed Methods Study
Source: JMIR Diabetes. 2019 Aug 6;4(3):e13724. doi: 10.2196/13724 (PMC6701161; doi:10.2196/13724)
Supplement: Multimedia Appendix 2 [file diabetes_v4i3e13724_app2.pdf]

Multimedia Appendix 2. Survey Responses for Usability and Acceptability of the mDAD Subdomain.

	Extremely Likely n(%)	Likely n(%)	Neutral n(%)	Unlikely n(%)	Extremely Unlikely n(%)
I would be interested in visiting the mDAD site again in the future.	21(64%)	12(36%)	0%	0%	0%
	Excellent	Very Good	Good	Fair	Poor
Overall, the quality of the mDAD site was …	58% (n=19)	39% (n=13)	3% (n=1)	0%	0%
	Strongly Agree n(%)	Agree n(%)	Neither Agree or Disagree n(%)	Disagree n(%)	Strongly Disagree n(%)
The information on the mDAD site was useful to me.	18(55%)	12(36%)	3(9%)	0(0%)	0(0%)
Overall, the layout and design of the mDAD site was attractive and pleasing to the eye.	19(58%)	14(42%)	0(0%)	0(0%)	0(0%)
The mDAD site was well organized.	22(67%)	10(30%)	1(3%)	0(0%)	0(0%)
The videos and graphs helped explain or clarify the written information on the site.	19(58%)	14(42%)	0(0%)	0(0%)	0(0%)
	Always n(%)	Often n(%)	Sometimes n(%)	Rarely n(%)	Never n(%)
I was able to gain access to the mDAD site.	28 (85%)	3(9%)	1(3%)	1(3%)	(0)0%
The links on the mDAD site worked.	28(85%)	4(12%)	1(3%)	0(0%)	0(0%)
I was able to view the videos on the mDAD site.	28(85%)	4(12%)	1(3%)	0(0%)	0(0%)
I could find the information I was looking for on the mDAD site in a few clicks.	21(64%)	10(30%)	2(6%)	0(0%)	0(0%)
